# The Impact of Dental Midline on Asymmetric Faces: Perspective of Laypersons and Dentists

**DOI:** 10.3390/ijerph182412904

**Published:** 2021-12-07

**Authors:** Joana Meneses Martins, Liliana Gavinha Costa, Ana Lidia Carvalho, Maria Conceição Manso, Sandra Gavinha, Mariano Herrero-Climent, Blanca Ríos-Carrasco, Carlos Falcão, Paulo Ribeiro

**Affiliations:** 1 FCS, Universidade Fernando Pessoa, 4249-004 Porto, Portugal; joanamenesesmartins@gmail.com (J.M.M.); analidiafgdecarvalho@gmail.com (A.L.C.); 2 FP-I3ID, FCS, Universidade Fernando Pessoa, 4249-004 Porto, Portugal; lilianac@ufp.edu.pt (L.G.C.); sgavinha@ufp.edu.pt (S.G.); cfalcao@ufp.edu.pt (C.F.); ribeiropaulo1@gmail.com (P.R.); 3 FP-I3ID, FP-ENAS, FCS, Universidade Fernando Pessoa, 4249-004 Porto, Portugal; cmanso@ufp.edu.pt; 4 Porto Dental Institute, 4150-518 Porto, Portugal; dr.herrero@herrerocliment.com; 5 Department of Periodontology, Universidad de Sevilla, 41009 Seville, Spain

**Keywords:** prosthodontics, aesthetic, attractiveness, axial midline angulation, facial asymmetry, laypersons, dentists

## Abstract

Background: The objective was to determine if asymmetric facial features, nasal and chin deviations, affect the perception of attractiveness of a dental midline angulation, and if it is consistent among both dentists and laypeople. It was also analyzed if factors, such as the sex, age group of the participants and the dentist’s area of operation are relevant in their assessment. Methods: A cross-sectional study, approved by the Ethics Committee of Fernando Pessoa University. From a symmetrical facial model (SFM) image, a natural-looking asymmetrical face was created. Based on this asymmetric facial model six images were created, with different directions and degrees of inclination of the dental midline. In total, 236 laypersons and 242 dentists completed the online questionnaire where they rated the self-perception of attractiveness of the eight images (VAS scale from 0 to 10). Non-parametric comparisons (IBM© SPSS Statistics vs. 27.0, *p* < 0.05). Results: The results showed a significant difference in the perception of attractiveness between laypeople and dentists. This finding was consistent regarding every image, except for the SFM. The factors, sex of the people participating and dentist’s area of operation, seemed only to contribute to a significant difference in the perception when it came to the SFM. The perceived attractiveness of the images, for dentists and laypersons, did not differ by age group of the participant, apart from images 6 and 8. Conclusions: Dentists are more rigorous about dental midline inclinations than laypersons. The perception of attractiveness was affected by the age group and sex of the participants and the dentist’s area of operation.

## 1. Introduction

The face is the part of the human body from which we infer more information about others, such as: gender, identity, intentions, emotions, attractiveness, age or ethnicity [1]. Thus, facial attractiveness has important social consequences, and is fundamental in human interactions [2].

However, the assessment of facial beauty is essentially subjective [3], so over the centuries philosophers and scientists have tried to quantify beauty through numerical symmetries and proportions, dividing the face into quadrants or thirds [3,4].

It is assumed that facial symmetry is closely related to attractiveness. However, human faces are not bilaterally symmetrical [5]. All human faces are asymmetrical, and no subjective scientific criteria are available to distinguish normal from abnormal asymmetries; therefore, this cannot be analyzed using purely straight lines [6]. 

It is known that one of the predominant factors in facial expression is the smile, since having an attractive smile is crucial for the individual’s acceptance in our society, by influencing the initial impression in relationships [7].

A direct consequence of the concern with appearance led patients inspired by pretty faces and beautiful smiles to seek treatments to improve dentofacial esthetics [8,9]. 

Aesthetics then became an important aspect of dental practice as, until about the last two decades, dentists considered aesthetics much less important than function, structure and biology [10].

Thus, one of the most important factors in defining the attractiveness of a smile is symmetry, and a properly positioned midline contributes to the balance and harmony of tooth composition [11,12].

As early as 1973, Lombardi considered the dental midline as the most important focal point in the aesthetics of a smile [13].

The coordination of the facial and dental midline is essential for the appreciation of facial harmony and balance. Although a subtle change of the dental midline to facial asymmetry within normal limits is acceptable, significant discrepancies in midline angulation can be quite detrimental to dentofacial esthetics [14].

Thus, one of the factors examined in the esthetic evaluation is the relationship between the dental midline and the facial midline [15]. Therefore, several studies have been carried out to test how much the dental midline can deviate or slant from the facial midline, before it becomes aesthetically unacceptable [7,16,17,18].

According to Beyer and Lindauer, the assessment of the dental midline position can be complicated if other midline facial structures are not well aligned, but they did not find how these facial structures can interfere with the perception of deviations of the dental midline [19].

It is important to understand the role that facial structures play in the aesthetics of the smile, as some facial asymmetries, such as chin and nose deviations, interfere with the perception of the beauty of the smile. The facial characteristics of each patient must be taken into account when planning any orthodontic treatment or oral rehabilitation [20].

It is essential that the dentist knows what the best positioning of the dental midline is in patients with facial asymmetries who are going to undergo orthodontic treatments or oral rehabilitation, so that these asymmetries become less noticeable and greater facial attractiveness can be achieved.

The aim of this study was to assess how asymmetric facial features, nose and chin deviations, affect the perception of attractiveness of an angled dental midline, and whether the perception of attractiveness varies between laypersons and dentists. The study also analyzed whether the gender, age of the participants and the main area of activity of the dentists are relevant factors in this assessment.

## 2. Materials and Methods

### 2.1. Data Collection Tool

Data were collected for 4 months, from February to May 2021, through an online questionnaire, created in Google Forms exclusively for this study. It was aimed at dentists and people who did not have knowledge in the field of dentistry (laypeople), of both sexes and aged 18 years or over. Participants had access to the questionnaire by means of online dissemination, in specific places for this purpose (link online), and voluntarily agreed to answer the questionnaire.

The questionnaire consisted of two parts. Initially, sociodemographic questions (age group, gender, whether layperson or dentist and dentist’s main area of work), followed by another part in which 8 photographs were presented at random and in two different phases of the questionnaire. At first, participants were asked to carefully observe 8 images in a random sequence. Later, in a new visualization, they were asked to rate them according to their self-perception of attractiveness using a VAS scale from 0 to 10, in which a score of 0 represented “not attractive at all” and 10 represented “very attractive”. The test view (first visualization of the images) helped participants to use the extremes of the scale in the second phase, as they tended to score more towards the middle of the scale when they did not see the photographs for the first time [18].

Dentists were asked what their main area of operation is in order to assess whether the different areas to which they are dedicated lead to different perceptions of attractiveness among the group of dentists. Thus, the dentist group was divided, according to its main area of expertise, into 2 distinct groups: area of operation linked to aesthetics and area of operation not linked to aesthetics. The operating area group linked to aesthetics encompassed orthodontics, prosthodontics and restorative dentistry.

### 2.2. Images 

A color photograph of the front face of a Caucasian female was taken with a Nikon D750 DSLR camera (Nikon 105 mm F2.8G vr AF-s ED-if Nikkor lens, Nikon, Tokyo, Japan) and a standard black background at a distance of two meters. From this photograph, a symmetrical facial model was created (digitally) and the image was manipulated, according to the needs of the study, using the computer program Adobe Photoshop© (Adobe Inc, San Jose, California).

**Figure 1:**
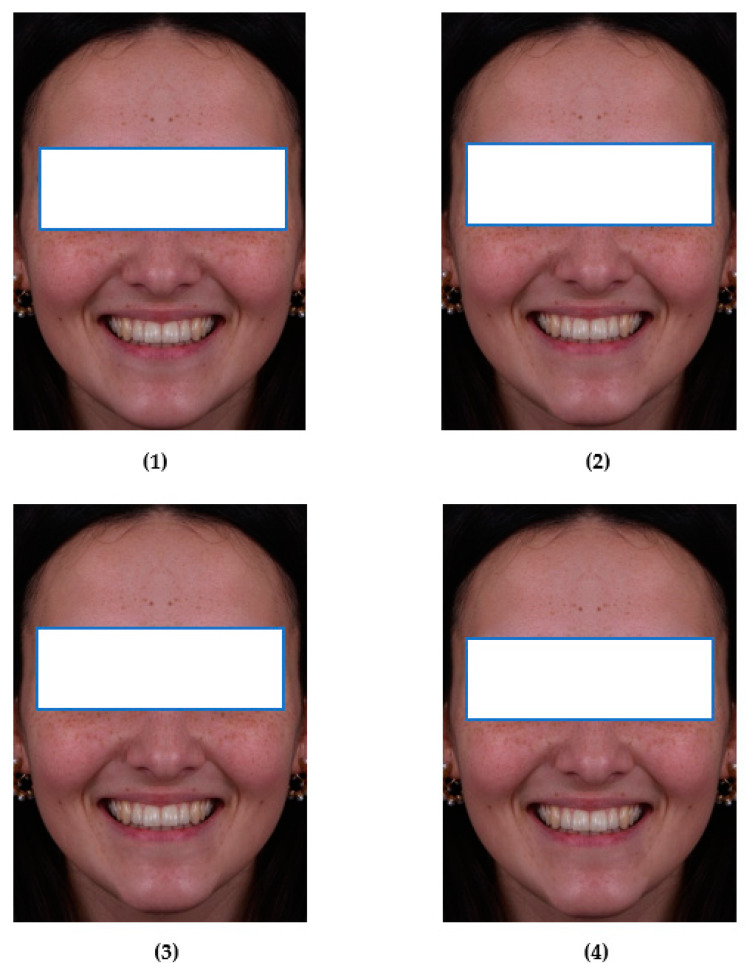

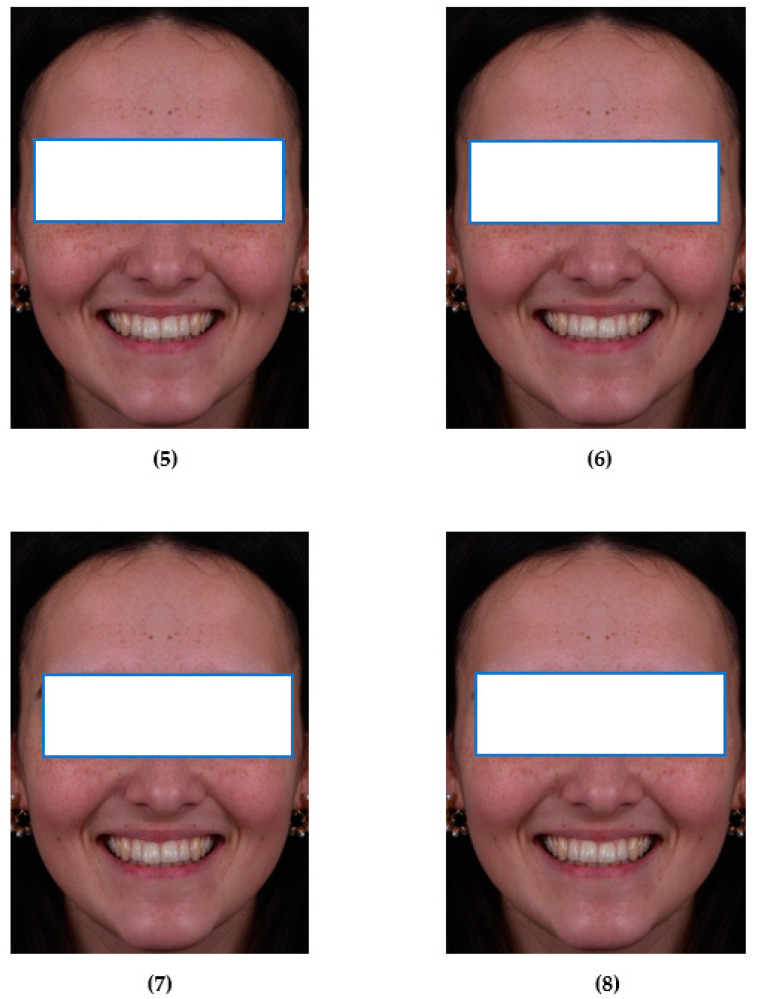
(**1**) Symmetrical facial model (SFM); (**2**) Asymmetric facial model (AFM); (**3**) AFM with dental midline inclination of 1.5° to the left; (**4**) AFM with dental midline inclination of 1.5° to the right; (**5**) AFM with dental midline inclination of 3.5° to the left; (**6**) AFM with dental midline inclination of 3.5° to the right; (**7**) AFM with dental midline inclination of 5° to the left; (**8**) AFM with dental midline inclination of 5° to the right.

From the symmetrical facial model, the nose and chin were displaced 3 mm to the left side of the face, in a parallel fashion, in order to create an asymmetrical, natural-looking face. Silva et al. (2013) demonstrated that 3 mm is the value that is below the visual recognition threshold [20]. The next step was to create 6 different versions of the asymmetric facial model, with three degrees of inclination of the dental midline 1.5°, 3.5° and 5°, both to the left and to the right. So, in total, 8 images were created (Figure 1).

### 2.3. Image Editing Process in Adobe Photoshop©

#### 2.3.1. Creation of a Symmetrical Facial Model (SFM)

The facial midline was correctly identified using the program’s ruler. The half of the image we would like to duplicate was selected (rectangular marquee tool). Then, after duplicating the half on a second layer, the images were mirrored to make a single fully symmetrical image. Finally, the two layers were joined to make a single final image.

#### 2.3.2. Creation of the Asymmetric Facial Model (AFM)

The nose was selected from the wings of the nose to the nasal bone (polygonal loop tool). Its horizontal displacement 3 mm to the left was performed, without altering its vertical position (free transformation and resizing).

The chin was selected in its entirety (polygonal loop tool). It was moved horizontally 3 mm to the left, without changing its vertical position (free transformation and resizing).

Color, texture, filling and luminosity adjustments were made to have a correct adaptation of the nose and chin in their new positions (polygonal lasso tool, content-sensitive filling, blending brush tool, eyedropper tool, history brush tool).

From the asymmetric facial model, 6 images were taken with an inclination of the dental midline.

#### 2.3.3. Creation of Images with Slanted Dental Midline (Images 3, 4, 5, 6, 7 and 8)

Six different versions of the asymmetric facial model were created, with three degrees of inclination of the dental midline 1.5°, 3.5° and 5°, both to the left and to the right.

The upper central incisors were selected (polygonal loop tool) and the rest of the asymmetrical image remained in the same place. The selected area was rotated by 1.5°, 3.5° and 5° (free transformation and rotation).

Color, texture, filling and luminosity adjustments were made to have a correct adaptation of the occlusal plane, gums and lips in their new positions (polygonal lasso tool, content-sensitive filling, mixing brush tool, eyedropper tool, history brush tool).

An additional adjustment was necessary for the discrepancy between the incisal edges of the maxillary central incisors, which was caused by the rotation. The longer incisal edge was refitted to be at the same height as the adjacent tooth (mixing brush tool).

### 2.4. Data Treatment

The data collected from the questionnaires were organized and stored in Excel (automatically) and the statistical analysis was performed using the IBM© SPSS Statistics vs. 27.0 (IBM Corp. released 2017, Armonk, NY, USA: IBM Corp.).

The information related to the perception of attractiveness (VAS scale) was described using the mean statistics and respective standard deviation, minimum and maximum, and the median and respective percentiles 25 and 75. The perception of attractiveness presented answers in all points of the scale (0 to 10), but it did not show a normal distribution of observations (Kolmogorov–Smirnov test), so the comparison of the center of the scale by the different groups was performed using non-parametric tests. The comparison of the degree of attractiveness of the 8 images was performed using the Friedman test for repeated measures and, upon detecting significant differences, the identification of which images differed was performed using the Wilcoxon test with Bonferroni correction. The Mann–Whitney U test was used to detect significant differences in the median of the perception of attractiveness of the VAS scale in the image choice (two groups: dentists or laypeople, gender, dentist’s area of operation), and the Kruskal–Wallis test was used to compare more than 2 groups (age group of participants).

The analysis was performed considering a significance level of 5%.

## 3. Results

A total of 478 adults participated in the study: 236 lay people (49.4%) and 242 Dentists (50.6%), most of them female (71%).

It was possible to verify in Table 1 that there are significant differences (*p* < 0.05) in the perception of attractiveness between laypeople and dentists, for all images, except for image 1 (SFM). In general, for images 2 to 8, dentists attribute, in average or median terms, a lower perception of attractiveness when compared to laypeople.

### 3.1. Comparison of Perception of Image Attractiveness between Groups (Dentists and Laypeople) 

According to the results in Table 1, in the lay group there are no statistically significant differences in the perception of attractiveness of images 1, 2, 3 and 4 (Wilcoxon test with Bonferroni correction, *p* > 0.05 for all comparisons). That is, no difference in attractiveness is detected even when there is an angulation of the dental midline up to 1.5°.

The perceptions of attractiveness of images 5, 6, 7 and 8 do not differ from each other (Wilcoxon test with Bonferroni correction, *p* > 0.05 for all comparisons), but they are significantly lower than those of images 1, 2, 3 and 4 (*p* ≤ 0.001 for all comparisons). This also means that, based on these images (5 to 8), laypeople do not perceive the attractiveness differently when the angulation of the dental midline occurs to the right or to the left.

As shown in Table 1, in the group of dentists, image 1 is the one with a higher median attractiveness perception when compared to the other images, thus there are statistically significant differences between image 1 and images 2 to 8 (Friedman test, *p* < 0.001). The perception of attractiveness between images 2, 3 and 4 do not differ, and although in images 3 and 4 there are already 1.5° dental midline inclinations, dentists do not perceive their attractiveness differently. Likewise, the attractiveness of images 3 and 4 does not significantly differ from image 5. However, image 5 differs significantly from image 2 (AFM). Image 5 is perceived as less attractive than image 2, just as image 5 differs significantly from image 6, with image 5 being considered more attractive than image 6. Image 6 differs significantly from image 7 and 8. The images with a dental midline inclination of 5° (images 7 and 8) are considered the least attractive. There are no statistically significant differences between them (7 and 8), with no difference in the perception of attractiveness when the angulation of the dental midline occurs to the right or to the left.

Through the analysis of Table 2, it can be concluded that there are no statistically significant differences between dentists with a practice area linked to aesthetics and those with a practice area not linked to aesthetics, for all images, except for image 1 (SFM), which is perceived as significantly more attractive by dentists with an area of expertise linked to aesthetics (*p* = 0.038, Mann–Whitney comparison test).

For dentists not linked to aesthetics (Table 2), there is no significant difference in the perception of median attractiveness between images 1 and 2 (Friedman test, *p* < 0.001). The perceptions of attractiveness between images 2, 3, 4 and 5 do not differ from each other, but from 3 to 5 they are less attractive than image 1; however, in images 3 and 4 there is already a 1.5° dental midline inclination and in image 5 there is already an inclination of the dental midline of 3.5° to the left. Likewise, the perception of attractiveness between images 6 and 7 does not differ significantly, and that of image 7 does not differ significantly from image 8 either, with images 7 and 8 being the less attractive.

### 3.2. Comparison of Perception of Image Attractiveness by Area of Operation of Dentists

As shown in Table 2, in dentists linked to aesthetics, image 1 is the one with the highest median attractiveness perception when compared to the other images, thus there are statistically significant differences between image 1 and images 2 to 8 (Friedman test, *p* < 0.001). The perceptions of attractiveness between images 2, 3, 4 and 5 do not differ from each other. Likewise, the perceptions of attractiveness between images 6 and 7 do not differ significantly, and image 7 does not differ significantly from image 8.

### 3.3. Comparison of Perception of Image Attractiveness by Sex in Each Group

As seen in Table 3, the perception of attractiveness of the images, both for dentists and for laypeople, does not differ by sex of the respondent, with the exception of image 1, which is perceived as significantly more attractive by women than by men (*p* = 0.047 and *p* = 0.026, respectively).

### 3.4. Comparison of Perception of Image Attractiveness by Age Group in Each Group 

According to Table 4, the perception of attractiveness of the images, both for dentists and for laypeople, does not differ by age group of the participant, with the exception of images 6 and 8.

Dentists > 45 years old perceive image 6 as being more attractive than dentists ≤35 years old and dentists between 36 and 45 years old (*p* = 0.032, Mann–Whitney multiple comparison test with Bonferroni correction, after Kruskal–Wallis test).

In image 8, significant differences are identified in the perception of median attractiveness by the age group of the participant, both for dentists and for laypeople. Dentists and laypeople aged >45 years perceive image 8 as being more attractive than those aged ≤35 years and 36 to 45 years (*p* = 0.005 and *p* = 0.049, respectively).

## 4. Discussion

The results showed that in cases where there were statistically significant differences between dentists and laypeople, dentists attributed a lower perception of attractiveness to all images, except for the SFM. It is therefore concluded that the group of dentists reflects being more demanding and rigorous in terms of attractiveness, possibly due to their academic training that enables them to recognize lighter dental midline inclinations. This is in line with other studies conducted in this regard, which have also shown that the group of dentists is more critical of the dental midline slope than the general public [7,16].

For dentists, any deviation from the SFM is considered less attractive, that is, as the degree of inclination of the dental midline increases, the image is perceived as less attractive. Other studies have already reached this conclusion, that increasing the dental midline slope decreases the attractiveness of a smile [7,16,17].

Dentists detected a difference in the perception of attractiveness between the image that is completely symmetrical (SFM) and the image that has only the deviation of the nose and chin (AFM). However, they did not detect differences between the image that has only the deviation of the nose and chin (AFM) and the images with a 1.5° dental midline inclination (images 3 and 4). This leads to the conclusion that the 1.5° dental midline inclination on an asymmetrical face is not sufficiently remarkable for dentists. This refers to the study by Kokich and colleagues, who found that dentists were able to identify discrepancies from 2 mm of dental midline angulation, considering them as visibly unattractive [16]. It should be noted that this study used linear measurements and photographs only of the smile.

When comparing the images with a 3.5° inclination of the dental midline, but in opposite directions (images 5 and 6), the dentists perceived a greater attractiveness in the image with a 3.5° inclination of the dental midline to the left (image 5), i.e., to the same side as the deviation of the nose and chin in the AFM. The fact that the dental midline slope was on the same side as the facial midline deviation could be considered more harmonious than when the dental midline was slanted to the opposite side. The same was found for laypeople by Silva et al., (2018) [18]; however, they did not analyze it for dentists.

The images with a 5° dental midline inclination (images 7 and 8) were considered the least attractive by dentists, with no significant differences between them; however, there was no difference in the perception of attractiveness when the dental midline angulation occurred right or left. Therefore, it seems that dentists consider 5° of dental midline inclination to be so unaesthetic that it is indifferent to them whether the dental midline inclination is on the same side as the deviation of the nose and chin or on the opposite side. Gul-e-Erum and Fida also demonstrated that 5° of dental midline angulation was perceived by dentists [17].

Based on the results, laypeople did not detect any difference in the perception of attractiveness between the image that is completely symmetrical (SFM) and the image that has only the deviation of the nose and chin (AFM), that is, it is possible to assume that the introduced asymmetries were not noticeable. This supports the study by Silva et al. who demonstrated that the 3mm displacement of the nose and chin is below the visual recognition threshold [20]. However, this did not happen in the group of dentists, as they were able to differentiate the completely symmetrical image (SFM) from the one with facial asymmetries (AFM), which leads us to believe that dentists are better at identifying facial asymmetries. Kokich et al. also demonstrated that dentists are more sensitive than laypeople to identifying deviations from what is considered ideal [16].

Laypeople did not observe differences between the SFM, AFM images and the images with a dental midline inclination of 1.5° (images 3 and 4), which may mean that dental midline inclinations up to 1.5° are unnoticeable by laypeople. In fact, no studies were found in the literature that analyzed the perception of attractiveness using a 1.5° dental midline angulation. Only Kokich et al. used in their study a dental midline angulation of 1 mm but concluded that laymen were only able to identify the dental midline discrepancy from 2 mm onwards [16]. In this study, 1.5° of dental midline inclination seems to be the visual recognition threshold for this group. Therefore, during the execution of orthodontic treatments or oral rehabilitation, there may be the possibility of an error margin of 1.5° of inclination of the dental midline until it becomes noticeable or unaesthetic for the patients. Consequently, this can also help in making decisions about the type of rehabilitative approach that should be implemented, as it can help the dentist to choose a more or less conservative treatment in order to meet the patient’s expectations.

The images with an inclination of the dental midline of 3.5° (images 5 and 6) and 5° (images 7 and 8) were considered the least attractive by laypeople and did not detect differences in the perception of attractiveness between them. Unlike dentists, laypeople do not perceive attractiveness differently when the dental midline angulation of 3.5° occurs to the right or to the left. Therefore, laypeople perceive it as equally unaesthetic whether there is a 3.5° or 5° inclination of the dental midline, and whether this inclination is on the same side as the deviation of the nose and chin or on the opposite side. This is an interesting finding as, in the study by Silva et al., despite also concluding that a dental midline with a 3.5° inclination can negatively impact the visual perception of facial beauty, they stated that this trend follows the direction that nose and chin asymmetries may be more attractive to laypeople, which is not consistent with this study [18]. These results are also in agreement with the research done by Gul-e-Erum and Fida (2008) who admitted that 5° of dental midline angulation is perceived by laypeople [17].

There were no statistically significant differences between dentists with the main area of operation linked to aesthetics and with the main area of operation not linked to aesthetics, for all images, except in the SFM (image 1). This image was perceived as more attractive by dentists with the main area of operation linked to aesthetics.

Unlike dentists with a main area of operation not linked to aesthetics, those with the main area of operation linked to aesthetics detected a difference in the perception of attractiveness between the image that is fully symmetrical (SFM) and the image that has only the deviation of the nose and chin (AFM). This presupposes that dentists with the main area of operation linked to aesthetics are more focused on symmetries and on the coincidence between the dental and facial midlines. Since, in the literature, there are only studies that normally only divide the practice area of dentists into orthodontists and generalist dentists, it is difficult to compare the results of this study with the others. However, the results found are in line with what was determined by Jackson et al., who studied the ability of orthodontists, generalist dentists and laypersons to assess facial symmetries and concluded that orthodontists demonstrated having a better experience in assessing facial symmetry than generalist dentists, but only in the most difficult cases [21].

However, both dentists with the main area of operation linked to aesthetics and dentists with the main area of operation not linked to aesthetics considered that images 2 to 5 were equally attractive. However, in image 2 there were no dental asymmetries, in images 3 and 4 there was already an inclination of the dental midline of 1.5° and in image 5 there was an inclination of the dental midline of 3.5° to the left. However, they considered that images 3,4 and 5 were less attractive than image 1. Likewise, the perception of attractiveness between images 6 and 7 did not differ significantly, and that of image 7 did not differ significantly from image 8, being considered as less attractive. When images with dental asymmetries were presented, the perception of attractiveness of dentists with the main area of operation linked to aesthetics did not differ from those not linked to aesthetics, which means that the former despite having a higher level of training in relation to the ideals of beauty and aesthetics, do not prove to be more rigorous or demanding. These results are opposed to what was found by Kokich et al., who divided the area of operation of dentists into orthodontists and generalist dentists and concluded that orthodontists were able to more easily identify a lower level of midline inclination than generalist dentists [16].

The perception of attractiveness of the images, either for dentists or for laypeople, did not differ by the sex of the respondent, except for image 1. This was perceived as significantly more attractive by females than by males, thus demonstrating that women have a better perception of what is completely symmetrical. These results are opposed to what was found in the studies by Thomas et al. and in Silva et al., in which the gender of the participants was not a factor to be considered in the perception of attractiveness [7,18].

The participant’s age group had an impact on the perception of attractiveness: participants aged >45 years did not seem to be as critical and demanding in relation to facial esthetics as participants aged ≤45 years. Dentists and laypersons aged >45 years perceived image 8 as being significantly more attractive than those ≤45 years old, and dentists aged >45 years also perceived image 6 as being significantly more attractive than the age group of ≤45 years. In these images, the inclination of the dental midline is to the right, that is, to the opposite side of the deviation of the nose and chin in the AFM, which does not appear to be as harmonious as when the deviation of the dental midline tends to follow the deviation of the nose and chin [18]. A possible explanation for this is that in the past the main demand for dental treatments was due to functional needs, while currently the focus is on dental aesthetics [10]. Perhaps, this is the reason why older participants perceive attractiveness differently from younger ones.

In addition to being essential for the dentist to know what the best positioning of the dental midline in patients with facial asymmetries is, it is equally important that they define an individualized treatment plan, before carrying out any type of aesthetic treatment that involves a positioning or alteration of the patient’s dental midline. Thus, the dentist will be able to ascertain whether the patient’s perception of attractiveness is similar to their own, as dentists are more critical of the dental midline slope than the general public [7,16], and perhaps the clinical detail perceived by dentists is not required by the patient.

### Limitations

In this study, there should have been a greater heterogeneity of the sample in relation to the age group and gender of the participants, as most participants were female and aged ≤35 years.

When comparing the results of this study with existing studies, it is difficult to establish objective comparisons, as each study differs in materials and methods and some studies do not sufficiently describe the methodology used.

Compared to this study, others used different measurement units to make the inclination of the dental midline (degrees/mm), and for some, despite using the same measurement units, their applicability in image processing generated different results: they used different groups of participants (laypersons/general dentists/orthodontists/art students); different areas of assessment (facial photographs/smile photographs); and different scales (Likert/VAS) to assess the perception of attractiveness.

For this reason, further studies are needed, in which there is not much variation in the methodology and in which there is greater sample heterogeneity, in order to be able to establish objective comparisons to understand what the true visual recognition threshold of a sloping midline on asymmetric faces is.

## 5. Conclusions

On asymmetric faces, the degree and direction of the inclination of the dental midline influence the perception of attractiveness of laypeople and dentists. 

Dentists reflect that they are more demanding and stricter in relation to the inclination of the dental midline than laypeople. 

Dentists perceived the fully symmetrical image as more attractive and as the degree of inclination of the dental midline increased, the images were perceived as less attractive.

Laypeople did not detect differences in the perception of attractiveness between images up to 1.5° of the dental midline inclination (images 1 to 4), nor between images of 3.5° and 5° (images 5 to 8).

Laypeople and dentists perceived an inclination of the dental midline from 3.5° as less attractive, which can harm facial attractiveness. However, if this trend follows the direction of nose and chin deviation, it is perceived as more attractive to dentists than following the opposite direction.

Dentists with a main area of operation linked to aesthetics seemed to identify facial symmetries better, but when dental asymmetries were presented, the perception of attractiveness in this group did not differ from those with a main area of operation not linked to aesthetics.

Female participants demonstrated to have a better perception of what is completely symmetrical than male participants.

Participants older than 45 years did not seem to be as critical and demanding in relation to facial aesthetics as participants aged under 45 years.

## Figures and Tables

**Table 1:** Comparison of perception of image attractiveness between groups (dentists and laypeople).

		Dentists (*n* = 242)	Laypeople (*n* = 236)	*p* *
Image 1	Mean ± SD	7.55 ± 1.93	7.36 ± 2.08	
	Min–Max	2–10	0–10	
	Median (P25–P75)	8 ^a^ (6–9)	8 ^a^ (6–9)	0.331
Image 2	Mean ± SD	6.45 ± 2.07	6.98 ± 2.16	
	Min–Max	0–10	0–10	
	Median (P25–P75)	7 ^b^ (5–8)	7 ^a^ (6–8)	0.004
Image 3	Mean ± SD	6.21 ± 2.1	6.97 ± 2.06	
	Min–Max	0–10	0–10	
	Median (P25–P75)	7 ^b,c^ (5–8)	7 ^a^ (6–8)	<0.001
Image 4	Mean ± SD	6.09 ± 2.17	6.94 ± 2.08	
	Min–Max	0–10	0–10	
	Median (P25–P75)	6 ^b,c^ (5–8)	7 ^a^ (6–8)	<0.001
Image 5	Mean ± SD	5.94 ± 2.14	6.37 ± 2.05	
	Min–Max	0–10	0–10	
	Median (P25–P75)	6 ^c^ (5–7)	7 ^b^ (5–8)	0.036
Image 6	Mean ± SD	5.4 ± 2.18	6.37 ± 2.15	
	Min–Max	0–10	0–10	
	Median (P25–P75)	6 ^d^ (4–7)	7 ^b^ (5–8)	<0.001
Image 7	Mean ± SD	4.89 ± 2.2	6.16 ± 2.18	
	Min–Max	0–10	0–10	
	Median (P25–P75)	5 ^e^ (4–7)	6 ^b^ (5–8)	<0.001
Image 8	Mean ± SD	4.63 ± 2.29	5.97 ± 2.29	
	Min–Max	0–10	0–10	
	Median (P25–P75)	5 ^e^ (3–6)	6 ^b^ (5–8)	<0.001
*p* **		<0.001	<0.001	

* T. U Mann–Whitney for group comparison; ** Friedman test for image comparison (in each group of participants). ^a,b,c,d,e^—different letters identify significant differences in the median value of perceived attractiveness within the groups (“a” higher, “b” lower, …“e” lower of all), according to the Wilcoxon comparison test with Bonferroni correction.

**Table 2:** Comparison of perception of image attractiveness by area of operation of dentists.

		Operating Area Linked to Aesthetics		*p* *
		**No** **(*n* = 158)**	**Yes** **(*n* = 84)**	
Image 1	Mean ± SD	7.35 ± 2.01	7.92 ± 1.72	
	Min–Max	2–10	2–10	
	Median (P25–P75)	8 ^a^ (6–9)	8 ^a^ (7–9)	0.038
Image 2	Mean ± SD	6.45 ± 2.04	6.46 ± 2.14	
	Min–Max	0–10	1–10	
	Median (P25–P75)	7 ^a,b^ (5–8)	7 ^b^ (5–8)	0.821
Image 3	Mean ± SD	6.21 ± 1.98	6.23 ± 2.31	
	Min–Max	1–10	0–10	
	Median (P25–P75)	6 ^b^ (5–8)	7 ^b,c^ (5–8)	0.674
Image 4	Mean ± SD	6.08 ± 2.19	6.12 ± 2.16	
	Min–Max	0–10	0–10	
	Median (P25–P75)	6 ^b^ (5–8)	6.5 ^b,c^ (5–8)	0.751
Image 5	Mean ± SD	5.94 ± 2.09	5.95 ± 2.25	
	Min–Max	0–10	0–10	
	Median (P25–P75)	6 ^b^ (5–7)	6 ^b,c^ (5–8)	0.832
Image 6	Mean ± SD	5.28 ± 2.08	5.63 ± 2.35	
	Min–Max	0–10	0–10	
	Median (P25–P75)	5 ^c^ (4–7)	6 ^c,d^ (4–7)	0.114
Image 7	Mean ± SD	4.85 ± 2.16	4.95 ± 2.29	
	Min–Max	0–10	0–10	
	Median (P25–P75)	5 ^c,d^ (4–7)	5 ^d,e^ (3–7)	0.626
Image 8	Mean ± SD	4.63 ± 2.2	4.62 ± 2.46	
	Min–Max	0–10	0–10	
	Median (P25–P75)	5 ^d^ (3–6)	5 ^e^ (3–7)	0.899
*p* **		<0.001	<0.001	

* T. U Mann–Whitney for group comparison; ** Friedman test for image comparison (in each group of participants). ^a,b,c,d,e^—different letters identify significant differences in the median value of perceived attractiveness within the groups (“a” higher, “b” lower, …“e” lowest of all), according to the Wilcoxon comparison test with Bonferroni correction.

**Table 3:** Comparison of perception of image attractiveness by sex, in each group.

		Dentists			Laypeople		
		F	M	*p* *	F	M	*p* *
Image 1	Mean ± SD	7.7 ± 1.94	7.29 ± 1.89		7.45 ± 2.08	7.06 ± 2.06	
	Min–Max	2–10	2–10		0–10	0–10	
	Median (P25–P75)	8 ^a^ (7–9)	8 ^b^ (6–9)	0.047	8 ^a^ (6–9)	7 ^b^ (6–8)	0.026
Image 2	Mean ± SD	6.59 ± 2.16	6.22 ± 1.88		7.05 ± 2.14	6.76 ± 2.23	
	Min–Max	1–10	0–10		0–10	1–10	
	Median (P25–P75)	7 (5–8)	6 (5–8)	0.056	7 (6–9)	7 (5.75–8)	0.674
Image 3	Mean ± SD	6.24 ± 2.14	6.17 ± 2.02		7.03 ± 2.05	6.76 ± 2.13	
	Min–Max	1–10	0–10		0–10	1–10	
	Median (P25–P75)	7 (5–8)	6 (5–8)	0.520	7 (6–8.25)	7 (5.75–8)	0.473
Image 4	Mean ± SD	6.15 ± 2.24	5.98 ± 2.07		7.04 ± 2.09	6.61 ± 2.03	
	Min–Max	0–10	0–10		0–10	0–10	
	Median (P25–P75)	6 (5–8)	6 (5–7)	0.555	7 (6–9)	7 (5–8)	0.491
Image 5	Mean ± SD	6.08 ± 2.16	5.69 ± 2.11		6.36 ± 2.12	6.39 ± 1.82	
	Min–Max	0–10	0–10		0–10	2–10	
	Median (P25–P75)	6 (5–8)	6 (5–7)	0.236	7 (5–8)	7 (5–7)	0.455
Image 6	Mean ± SD	5.55 ± 2.28	5.14 ± 1.97		6.36 ± 2.17	6.41 ± 2.09	
	Min–Max	0–10	0–9		0–10	0–10	
	Median (P25–P75)	6 (4–7)	5 (4–7)	0.353	6 (5–8)	7 (5–8)	0.949
Image 7	Mean ± SD	4.84 ± 2.25	4.98 ± 2.11		6.13 ± 2.16	6.26 ± 2.28	
	Min–Max	0–10	0–9		0–10	1–10	
	Median (P25–P75)	5 (3–7)	5 (4–7)	0.942	6 (5–8)	6.5 (5–8)	0.399
Image 8	Mean ± SD	4.65 ± 2.38	4.6 ± 2.12		5.86 ± 2.28	6.37 ± 2.29	
	Min–Max	0–10	0–10		0–10	0–10	
	Median (P25–P75)	5 (3–6)	5 (3–6)	0.298	6 (4–7.25)	7 (5–8)	0.098

* T. U Mann–Whitney for group comparison; ^a,b^—different letters identify significant differences in the median value of perceived attractiveness between genders (“a” higher and “b” lower), according to the Mann–Whitney comparison test.

**Table 4:** Comparison of perception of image attractiveness by age group in each group.

		Dentists				Laypeople			
		≤35 Years	36–45 Years	>45 Years	*p*	≤35 Years	36–45 Years	>45 Years	*p*
		(*n* = 145)	(*n* = 60)	(*n* = 37)		(*n* = 172)	(*n* = 15)	(*n* = 49)	
Image 1	Mean ± SD	7.72 ± 1.92	7.27 ± 1.89	7.32 ± 1.99		7.47 ± 2.02	7.27 ± 2.6	7± 2.09	
	Min–Max	2–10	2–10	2–10		1–10	0–10	0–10	
	Median (P25–P75)	8 (6.5–9)	7.5 (6–9)	8 (6–9)	0.144	8 (6–9)	8 (5–9)	7 (6–8)	0.327
Image 2	Mean ± SD	6.34 ± 2.19	6.5 ± 1.96	6.84 ± 1.71		7.1 ± 2.15	5.87 ± 2.56	6.9 ± 1.98	
	Min–Max	0–10	1–10	3–9		0–10	1–9	0–10	
	Median (P25–P75)	7 (5–8)	7 (5–8)	7 (5.5–8)	0.536	7 (6–9)	7 (4–8)	7 (6–8)	0.163
Image 3	Mean ± SD	6.06 ± 2.17	6.2 ± 1.9	6.86 ± 2.03		7.01 ± 2.05	6.33 ± 2.61	7 ± 1.95	
	Min–Max	0–10	2–10	1–10		0–10	1–10	0–10	
	Median (P25–P75)	6 (4.5–8)	7 (5–8)	7 (5.5–8)	0.087	7 (6–8)	7 (5–8)	7 (6–8)	0.571
Image 4	Mean ± SD	5.99 ± 2.21	5.88 ± 2.11	6.84 ± 2.05		6.98 ± 2.04	6.27 ± 2.87	7 ± 1.97	
	Min–Max	0–10	0–10	2–10		0–10	0–10	0–10	
	Median (P25–P75)	6 (5–8)	6 (5–7)	7 (5.5–8.5)	0.090	7 (6–8)	7 (3–8)	7 (6–8)	0.794
Image 5	Mean ± SD	5.97 ± 2.25	5.7 ± 1.88	6.24 ± 2.14		6.26 ± 2.05	6.47 ± 2.23	6.73 ± 1.97	
	Min–Max	0–10	1–10	2–10		0–10	2–10	0–10	
	Median (P25–P75)	6 (5–8)	6 (5–7)	7 (5–8)	0.385	7 (5–7)	6 (5–8)	7 (6–8)	0.253
Image 6	Mean ± SD	5.3 ± 2.2	5.15 ± 2.17	6.24 ± 1.94		6.31 ± 2.09	5.73 ± 2.71	6.76 ± 2.17	
	Min–Max	0–10	0–10	2–9		0–10	0–10	0–10	
	Median (P25–P75)	5 ^b^ (4–7)	5 ^b^ (4–7)	6 ^a^ (5–8)	0.032	6.5 (5–8)	5 (4–8)	7 (6–8)	0.169
Image 7	Mean ± SD	4.74 ± 2.2	4.8 ± 2.09	5.62 ± 2.28		6.07 ± 2.19	5.73 ± 2.6	6.59 ± 1.98	
	Min–Max	0–10	0–10	2–9		0–10	1–10	0–10	
	Median (P25–P75)	5 (3–7)	5 (4–6)	6 (3.5–8)	0.116	6 (5–8)	6 (4–7)	7 (5–8)	0.249
Image 8	Mean ± SD	4.32 ± 2.21	4.65 ± 2.19	5.78 ± 2.42		5.79 ± 2.32	5.73 ± 2.58	6.69 ± 1.96	
	Min–Max	0–10	0–10	1–10		0–10	0–9	0–10	
	Median (P25–P75)	4 ^b^ (3–6)	5 ^a,b^ (3–6)	6 ^a^ (4–8)	0.005	6 ^b^ (4–8)	6 ^a,b^ (3–8)	7 ^a^ (5–8)	0.049

^a,b^—different letters identify significant differences in the median value of perceived attractiveness (“a” higher and “b” lower), according to the Mann–Whitney multiple comparison test with Bonferroni correction, after the Kruskal–Wallis test.
